# Treatment of Respiratory Viral Coinfections

**DOI:** 10.3390/epidemiologia3010008

**Published:** 2022-02-23

**Authors:** Paul Alexander, Hana M. Dobrovolny

**Affiliations:** Department of Physics & Astronomy, Texas Christian University, Fort Worth, TX 76129, USA; pauldalexander21@gmail.com

**Keywords:** mathematical modeling, antiviral, drug efficacy, coinfection

## Abstract

With the advent of rapid multiplex PCR, physicians have been able to test for multiple viral pathogens when a patient presents with influenza-like illness. This has led to the discovery that many respiratory infections are caused by more than one virus. Antiviral treatment of viral coinfections can be complex because treatment of one virus will affect the time course of the other virus. Since effective antivirals are only available for some respiratory viruses, careful consideration needs to be given on the effect treating one virus will have on the dynamics of the other virus, which might not have available antiviral treatment. In this study, we use mathematical models of viral coinfections to assess the effect of antiviral treatment on coinfections. We examine the effect of the mechanism of action, relative growth rates of the viruses, and the assumptions underlying the interaction of the viruses. We find that high antiviral efficacy is needed to suppress both infections. If high doses of both antivirals are not achieved, then we run the risk of lengthening the duration of coinfection or even of allowing a suppressed virus to replicate to higher viral titers.

## 1. Introduction

The advent of multiplex PCR assays capable of detecting many different viruses in one patient sample [1] has led to the discovery that many patients (ranging from 20–40%) presenting with influenza-like illness are, in fact, infected with more than one virus [2,3]. A variety of respiratory viruses are capable of presenting as part of a coinfection including influenza (both A and B) [4], respiratory syncytial virus (RSV) [5,6,7], rhinovirus (hRV) [7,8], parainfluenza virus (PIV) [9], and human metapneumovirus (hMPV) [10]. Coinfections with the novel coronavirus have also been observed [11,12,13,14,15], thus strategies need to be developed in order to treat patients with these coinfections [16].

Given the variety in possible coinfection pairs, it has been difficult to determine the effect of coinfections on the severity of clinical outcomes, with some studies finding that coinfections are less severe than monoinfections [17,18], other studies finding that coinfections are more severe than monoinfections [19,20,21,22], and yet other studies finding no difference between mono- and co-infections [6,18,23,24,25,26]. Studies indicate that the time and order of initial inoculum of the two viruses can play a role in the severity of the resulting disease [27,28,29,30,31,32,33], with one virus often suppressing the replication of another. Unfortunately, we often do not know when the patient was infected with either virus, thus we cannot tell if viral suppression is occurring. In this case, antiviral treatment targeted at the dominant virus, without corresponding treatment of the suppressed virus might lead to a more severe infection with the suppressed virus. Such scenarios make it difficult to determine how doctors should approach treating coinfections.

Unfortunately, there is little guidance for physicians since studies of treatment of coinfections are limited. One retrospective study of influenza coinfections did not find any difference in outcomes between patients treated with an antiviral and those who were not [34]. Another retrospective study of patients presenting with bronchiolitis showed similar responses to treatment with inhaled adrenaline whether the bronchiolitis was caused by a single viral pathogen or a coinfection [35]. An assay system for testing of antivirals against influenza/RSV coinfections has been developed [36] and has been used to identify compounds that are effective against both viruses [36,37], though it does not appear that any have been tested clinically for coinfections yet. Another promising idea is the development of pan-antivirals capable of targeting several different viruses simultaneously, however these compounds are currently in the computational design phase and have not yet been tested [38].

In order to provide better clinical guidance, mathematical modeling can be used to investigate possible treatment strategies. Mathematical modeling with viral kinetics models has previously been used to help assess possible treatments for a variety of viral infections and has yielded actionable guidance for clinicians. Mathematical models have helped develop the triple-drug cocktail currently used for treatment of human immunodeficiency virus (HIV) [39,40] and have also been used to investigate combination therapy for influenza [41,42,43], hepatitis C [44,45], chikungunya virus [46], and dengue [47]. Models have also been used to determine effective treatment regimens for zika virus [48], hepatitis B [49], hepatitis C [50], HIV [51], and influenza [52,53] by examining possible doses and timings to determine minimum doses required to suppress infection. In the current SARS-CoV-2 pandemic, mathematical models were used to investigate the possibility of re-purposing existing antivirals to treat SARS-CoV-2 [54,55,56]. The use of mathematical models in this context allows for an examination of more doses and treatment timings than is possible experimentally, focusing future pre-clinical and clinical antiviral trials on treatment regimens that are most likely to succeed.

In this paper, we use mathematical models to study the effect of treatment on viral coinfections. We assume that antivirals are available for both infections, can be taken and dosed independently, and do not interact. We find that for certain combinations of antiviral drug efficacy, treatment can lead to longer lasting coinfections, suggesting that care must be taken in deciding whether or not to treat coinfected patients.

## 2. Materials and Methods

### 2.1. Viral Kinetics Models

We use two previously published mathematical models of viral coinfections [57,58]. The first model is based on the assumption that viruses interact only through competition of the viruses for target cells,

(1) $$\begin{array}{cc} \mathrm{Target}\;\mathrm{cells}:\;\dot{T} & =-\sum_{i}\beta_{i}TV_{i}\\ \mathrm{Eclipse}\;\mathrm{cells}:\;\dot{E_{i}} & =\beta_{i}TV_{i}-k_{i}E_{i}\\ \mathrm{Infected}\;\mathrm{cells}:\;\dot{I_{i}} & =k_{i}E_{i}-\delta_{i}I_{i}\\ \mathrm{Virus}:\;\dot{V_{i}} & =p_{i}I_{i}-c_{i}V_{i}. \end{array}$$

In the model, target cells (*T*) are infected by either virus at rate $\beta_{i}$ (i=1,2). The cells then enter the eclipse phase ($E_{i}$) where the virus is replicating inside the cells, but not yet producing virus. The cells move from the eclipse phase to the infectious phase ($I_{i}$) at rate $k_{i}$ and die at rate $\delta_{i}$. Virus is produced at rate $p_{i}$ by infectious cells and virus is cleared at rate $c_{i}$. This model results in only acute coinfections.

The second model includes cell regeneration and allows for both viruses to infect the same cell (superinfection) [58]. This model allows for the possibility of chronic coinfections. Since chronic infections might respond differently to treatment, we use the model with superinfection to investigate treatment of these types of coinfection,

(2) $$\begin{array}{cc} \mathrm{Target}\;\mathrm{cells}:\;\dot{T} & =r-aT-\sum_{i}\beta_{i}TV_{i}\\ \mathrm{Eclipse}\;\mathrm{cells}:\;\dot{E_{i}} & =\beta_{i}TV_{i}-k_{i}E_{i}-\beta_{ji}E_{i}V_{j}\\ \mathrm{Superinfected}\;\mathrm{eclipse}\;\mathrm{cells}:\;\dot{E_{3}} & =\sum_{i,j}\beta_{ji}E_{i}V_{j}-k_{3}E_{3}\\ \mathrm{Infectious}\;\mathrm{cells}:\;\dot{I_{i}} & =k_{i}E_{i}-\delta_{i}I_{i}\\ \mathrm{Superinfected}\;\mathrm{infectious}\;\mathrm{cells}:\;\dot{I_{3}} & =k_{3}E_{3}-\delta_{3}I_{3}\\ \mathrm{Virus}:\;\dot{V_{i}} & =p_{i}I_{i}+p_{ij}I_{3}-c_{i}V_{i}. \end{array}$$

This model is similar to the previous model with the exception that we now include superinfected cells ($E_{3}$ and $I_{3}$) that are infected with both viruses. Note that the model assumes that superinfection can only happen when a cell is in the eclipse phase—once a cell starts producing a virus of one type, it can no longer become infected with the second virus.

### 2.2. Modeling Drug Effect

We model the effect of a drug using the drug efficacy, a number between 0 and 1, that is related to the drug concentration through the $E_{\max}$ model [59],

(3) $$\varepsilon =\frac{\varepsilon_{\max}D}{D+{\mathrm{IC}}_{50}},$$

where *D* is the drug concentration, $\varepsilon_{\max}$ is the maximum effect of the drug, and ${\mathrm{IC}}_{50}$ is the concentration at which half the maximum effect is achieved.

In this study, we are not modeling specific antivirals, but choose to focus on two possible mechanisms of action that are used by several antivirals. We model a drug that blocks infection by multiplying β by (1−ε). Examples of this type of antiviral include fusion inhibitors that block entry of the virus into the cell and have been developed for a number of respiratory viruses such as influenza [60,61], SARS-CoV-2 [62,63], RSV [64,65], PIV [66], and hMPV [67]. We also consider a drug that blocks viral production or release by multiplying *p* by (1−ε). This is used to model drugs such as neuraminidase inhibitors for influenza [68], remdesivir for SARS-CoV-2 [55,69], and favipiravir for Ebola and zika [48,70].

### 2.3. Simulations

Since a previous study found that coinfections of viruses with similar growth rates result in little change in the viral time course of either virus, while coinfections of viruses with different growth rates lead to viral block, we study the two different cases using two different pairs of viruses. Influenza A virus (IAV) and respiratory syncytial virus (RSV) are paired together because their growth rates are similar, so there is a fairly equal sharing of target cells and little change in viral time course when the two occur in a coinfection. Parainfluenza virus (PIV) and human rhinovirus (hRV) are paired together because PIV’s growth rate is much slower than hRV, thus PIV growth will be inhibited in the presence of hRV. The parameter values for all four viruses are taken from [57] and are given in Table 1.

For the superinfection model, we assume that the infection rate for superinfecting a cell is the same as the infection rate for infecting target cells. For example, the infection rate for influenza is the same whether it is infecting an uninfected cell or a cell that is already infected with RSV. We also assume that the production rates for singly infected cells and superinfected cells are the same. For IAV/RSV simulations, we use the eclipse transition rate and infectious cell death rate for IAV as the eclipse transition rate and infectious cell death rate of superinfected cells. For PIV/hRV simulations, we use the eclipse transition rate and infectious cell death rate of hRV as the eclipse transition rate and infectious cell death rate of superinfected cells. We also set the cell regeneration rate to r=0.03cells/d [71,72], and the natural cell death rate at a=0.03/d to maintain an assumption of baseline homeostatic conditions.

We also explored the effect of mechanism of action of the antivirals. Since we are including two possible antiviral mechanisms, there are four combinations of treatments: (i) Both viruses are treated with a drug that blocks viral entry, (ii) both viruses are treated with a drug that blocks viral production, (iii & iv) one virus is treated with a drug that blocks viral entry, and the other with a drug that blocks viral production and the reverse.

The differential equations were solved using the odeint function in python’s scipy package. To assess how well the drugs treated coinfections, we measured the duration of each virus infection individually, the co-infection durations, and the maximum viral loads for each virus. We present these measurements as measurements relative to the untreated value, i.e., $A_{\mathrm{treated}}/A_{\mathrm{untreated}}$, where *A* is the characteristic being measured, such that a value of 1 indicates no change due to treatment, a value larger than 1 indicates an increase due to treatment, and a value less than 1 indicates a decrease due to treatment.

## 3. Results

We use two previously published mathematical models of viral coinfection. The first model assumes the two viruses compete for the resource of target cells [57], while the second assumes that the two viruses can simultaneously infect a single cell [58]. The equations for both models are described in the Methods section. For both models, we examine two extreme cases of interacting viruses. The first uses the example of influenza (IAV) and respiratory syncytial virus (RSV), which have similar growth rates [57]. A coinfection with these viruses does not substantially reduce the viral load of either virus. The second uses the example of parainfluenza (PIV) and rhinovirus (hRV). Rhinovirus has a much larger growth rate than PIV [57], so in a coinfection, PIV viral loads are suppressed and hRV viral loads remain essentially unchanged from an hRV-only infection.

### 3.1. Basic Coinfection Model

**Figure 1:**
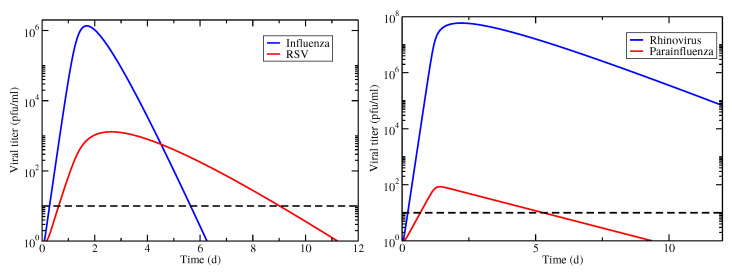
Viral titer time courses for coinfections involving IAV/RSV (**left**) and hRV/PIV (**right**). Infections begin simultaneously with equal amounts of virus. The horizontal dashed line indicates the threshold used for determining infection durations.

We first consider these two examples under the assumption that the viruses are competing for target cells (model Equation (1)). Viral titer time courses for these two cases are shown in Figure 1. From the time courses, we see that coinfection duration in the IAV/RSV coinfection is determined by both RSV and IAV time courses (RSV determines the start time and IAV determines the end time), while the coinfection duration for hRV/PIV coinfection is determined solely by the duration of the PIV infection.

#### 3.1.1. Viruses with Similar Growth Rates: Influenza and RSV

**Figure 2:**
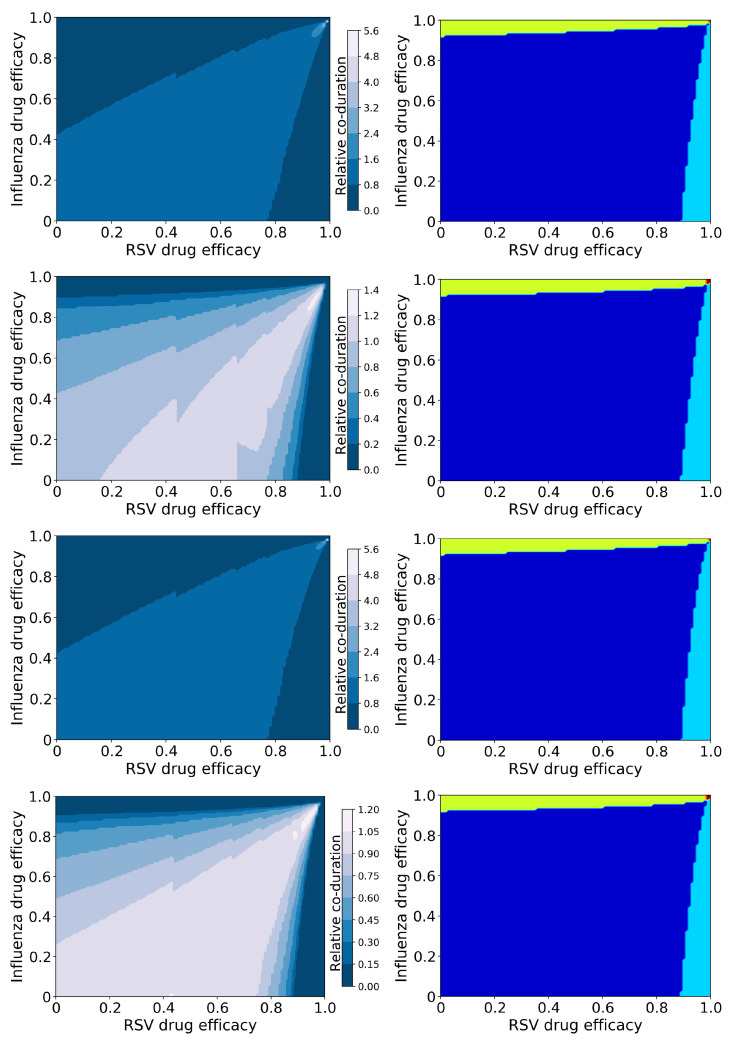
Coinfection durations and outcomes for treated influenza and RSV coinfections. (**Left column**): Figures show the coinfection duration relative to an untreated coinfection where a value of 1 indicates that treatment has not changed the coinfection duration. (**Right column**): Figures show regions of different treatment outcomes: Dark blue indicates that both infections are detectable; light blue indicates that only IAV is detectable; yellow indicates that only RSV is detectable; and red indicates that both infections are suppressed. Figures show treatment with both antivirals reducing the infection rate (**top row**); the influenza antiviral reducing infection rate and the RSV antiviral reducing viral production (**second row**); the influenza antiviral reducing viral production and the RSV antiviral reducing infection rate (**third row**); and both antivirals reducing viral production (**bottom row**).

Figure 2 shows the relative coinfection durations for treated IAV/RSV coinfections along with maps of treatment outcomes. The drug efficacy for each antiviral varies from 0 to 1 on each axis with the resulting relative coinfection duration given by the color (left column). In the right column, diagrams indicate regions where treatment does not suppress either infection (dark blue); treatment suppresses the RSV infection, but not influenza infection (light blue); treatment suppresses influenza infection, but not RSV infection (yellow); and treatment suppresses both infections (red). The figure shows treatment with all four combinations of antivirals, as described in Methods. Figures showing the durations of IAV and RSV, as well as the peak viral loads for both viruses, are included in the  Supplementary Material (Figures S1–S4).

There is a clear difference in the coinfection durations based on mechanism of action of the antiviral—the figures in the first and third rows assume the RSV antiviral reduces infection rate, while those in the second and fourth assume the RSV antiviral reduces viral production. The mechanism of action of the influenza antiviral also changes the predicted coinfection duration, however to a lesser degree. In all cases, it is possible for treatment to lengthen the duration of the coinfection; when the RSV antiviral reduces viral production, the increase in coinfection duration is slight (a maximum of 1.4 times longer than untreated), however when the RSV antiviral reduces infection, the increase in coinfection duration is significant (5.6 times longer than untreated). The longest coinfection durations occur when both antiviral efficacies are high (about 0.98 or 0.99), indicating that treatment of coinfections with a slightly suboptimal dose could potentially lengthen the coinfections.

While there are differences in coinfection duration of treated infections caused by different mechanisms of action, the broad treatment outcomes (suppression of infection) are independent of the mechanism of action (Figure 2 (right column)). There are two regions where the coinfection duration goes to zero: Where the efficacy of the RSV antiviral is high, and where the influenza antiviral efficacy is high. RSV is suppressed (RSV duration and viral peak go to zero,  Figures S2 and S4) with high RSV antiviral efficacy, however if the influenza antiviral dose is not high enough, influenza duration and viral titer peak actually increase (Figures S1 and S3). We see a similar effect when the influenza antiviral efficacy is high enough to suppress influenza, however the RSV antiviral efficacy is not high enough to suppress RSV. Thus a coinfection duration of zero does not indicate a complete cure since one of the infections can still remain; there is only a very small range of high drug efficacies of both antivirals where both infections will be suppressed.

#### 3.1.2. Viruses with Different Growth Rates: hRV and PIV

**Figure 3:**
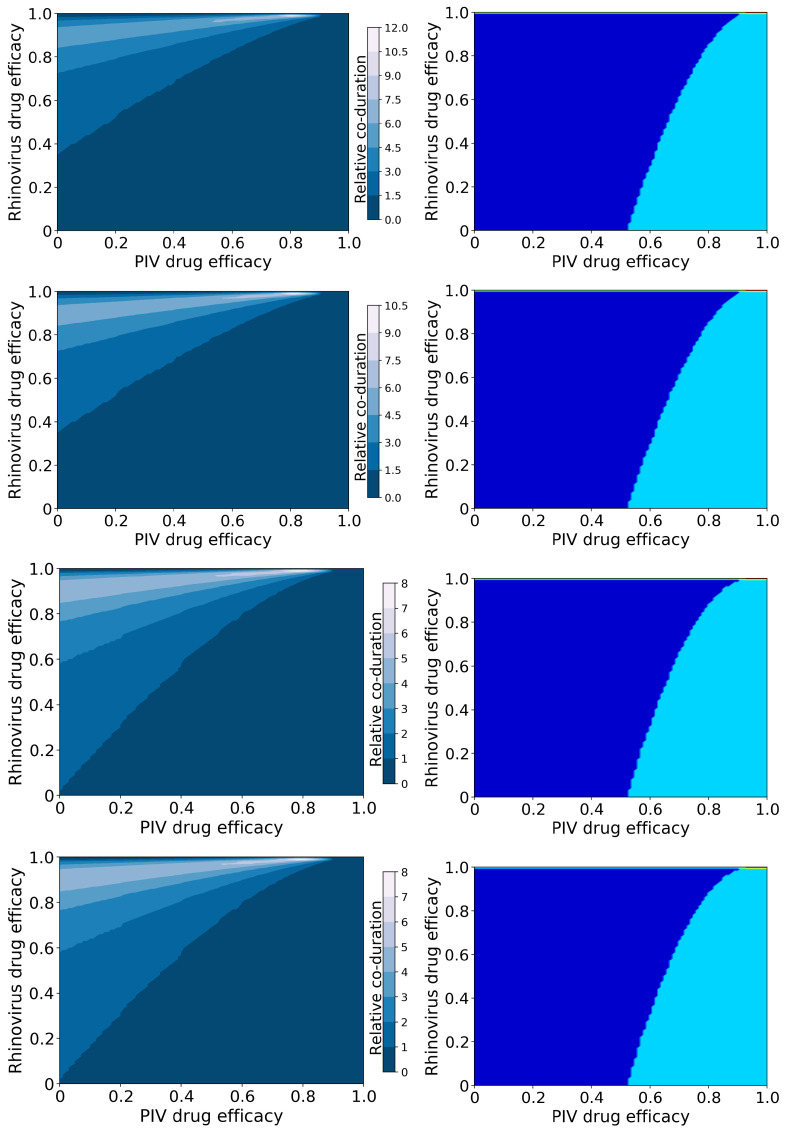
Coinfection durations and outcomes for treated rhinovirus and PIV coinfections. (**Left column**): Figures show the coinfection duration relative to an untreated coinfection where a value of 1 indicates that treatment has not changed the coinfection duration. (**Right column**): Figures show regions of different treatment outcomes: Dark blue indicates that both infections are detectable; light blue indicates that only hRV is detectable; yellow indicates that only PIV is detectable; and red indicates that both infections are suppressed. Figures show treatment with both antivirals reducing infection rate **(top row**); the rhinovirus antiviral reducing infection rate, and the PIV antiviral reducing viral production (**second row**); the rhinovirus antiviral reducing viral production and the PIV antiviral reducing infection rate (**third row**); and both antivirals reducing viral production (**bottom row**).

Figure 3 shows the relative coinfection durations and treatment outcomes for treated hRV/PIV coinfections. Figures showing the durations of hRV and PIV, as well as the peak viral loads, are included in the Supplementary Material (Figures S5–S8).

In this case, we again see a change in the amount of coinfection duration increase that depends on the mechanism of action, however it now depends on the mechanism of action of the rhinovirus antiviral. When the hRV antiviral reduces infection, we see a much larger increase in coinfection duration (up to 12 times longer than untreated) than when the hRV antiviral reduces viral production (up to 8 times longer than untreated). The large increase in coinfection duration occurs because hRV blocks replication of PIV in an untreated infection. When we treat the hRV infection, PIV has access to more target cells, so the PIV infection duration and viral titer peak both increase (Figures S6 and S8). Since the duration of the PIV infection determines the coinfection duration, treating the hRV without treating PIV leads to an increased coinfection duration.

The possible treatment outcomes for this coinfection differ from the treatment outcomes for IAV/RSV infections. In the case of hRV/PIV coinfection, there is almost no antiviral treatment combination that will lead to the suppression of hRV and only a PIV infection remaining. We also see a much broader range of dose combinations that will lead to complete suppression of PIV, which is not surprising since PIV was already naturally suppressed by hRV. Note that both antivirals need to be essentially 100% effective in this case to achieve a full cure of both infections.

### 3.2. Superinfection Model

**Figure 4:**
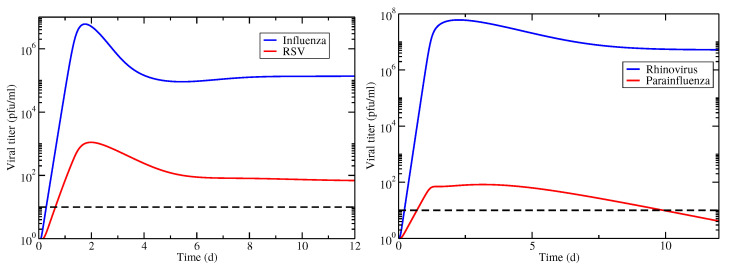
Viral titer time courses for coinfections with superinfection involving IAV/RSV (**left**) and hRV/PIV (**right**). Infections are started simultaneously with equal amounts of virus. The horizontal dashed line indicates the threshold used for determining infection durations.

We examine the same two cases of interacting viruses using the superinfection model (Equation (2)). The viral titer time courses for both virus combinations are shown in Figure 4. In this case, the untreated infections result in chronic coinfections, although in the case of hRV/PIV coinfection, the PIV chronic load is below the threshold of detection, therefore this would not be identified as a chronic coinfection. Note that this means that hRV/PIV coinfection has a finite coinfection duration in this case, however the IAV/RSV coinfection does not.

#### 3.2.1. Viruses with Similar Growth Rates: Influenza and RSV

**Figure 5:**
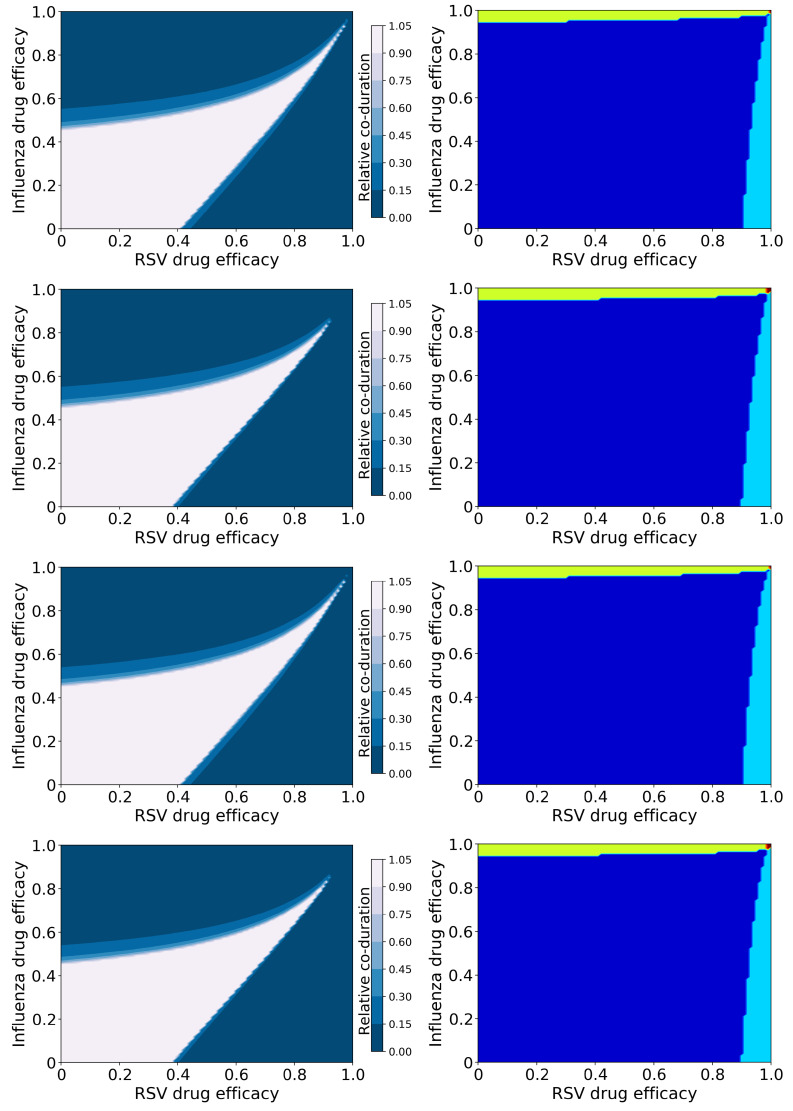
Coinfection durations and outcomes for treated influenza and RSV coinfections. (**Left column**): Figures show the coinfection duration relative to an untreated coinfection where a value of 1 indicates that treatment has not changed the coinfection duration. (**Right column**): Figures show regions of different treatment outcomes: Dark blue indicates that both infections are detectable; light blue indicates that only IAV is detectable; yellow indicates that only RSV is detectable; and red indicates that both infections are suppressed. Figures show treatment with both antivirals reducing infection rate (**top row**); the influenza antiviral reducing infection rate, and the RSV antiviral reducing viral production (**second row**); the influenza antiviral reducing viral production and the RSV antiviral reducing infection rate (**third row**); and both antivirals reducing viral production (**bottom row**).

Figure 5 shows coinfection durations (left column) and treatment outcomes (right column) for IAV/RSV chronic coinfections as functions of the antiviral efficacies. Infection durations and viral titer peaks for IAV and RSV individually are included in the Supplementary Material (Figures S9–S12).

Since the untreated coinfection is chronic, treatment does not run the risk of lengthening the infection, as was seen with the previous model. However, there is a wide range of antiviral drug efficacies that will not shorten the coinfection (white band starting from the bottom left corner of the duration graphs). There is some slight variation in the size of this region based on mechanism of action of the antiviral, however mechanism of action does not have as strong an effect in this model as it did in the previous model.

Interestingly, the broad treatment outcomes (Figure 5), drug combinations for which different viruses are suppressed, predicted by this model are the same as those predicted by the basic model. Note that suppression of an infection, whether it is acute or chronic, depends on the basic reproduction number ($R_{0}$)—if the antiviral effect is large enough to drive $R_{0}$ below 1, the infection will be suppressed. In this case, the basic reproductive number for treated infections is:

$$R_{\mathrm{treated}}=\left(1-\epsilon_{1}\right)\left(1-\epsilon_{2}\right)R_{0},$$

where $\epsilon_{1}$ and $\epsilon_{2}$ are the efficacies of the two antivirals, and $R_{0}$ is the basic reproductive number of the untreated infection (equations for $R_{0}$ for the basic model are found in [57] and for the superinfection model in [58]). Since we used the same parameter values for both infections, and set a=r, the basic reproductive number for each individual infection, as well as for the overall coinfection is the same for both models, thus the broad treatment outcomes are the the same for both models. Note that this equation is also independent of the mechanism of action of the antiviral, therefore suppression of infections is also independent of mechanism of action.

#### 3.2.2. Viruses with Different Growth Rates: hRV and PIV

Figure 6 shows coinfection durations (left column) and treatment outcomes (right column) for hRV/PIV chronic coinfections as functions of the antiviral efficacies. Infection durations and viral titer peaks for hRV and PIV individually are included in the Supplementary Material (Figures S13–S116).

**Figure 6:**
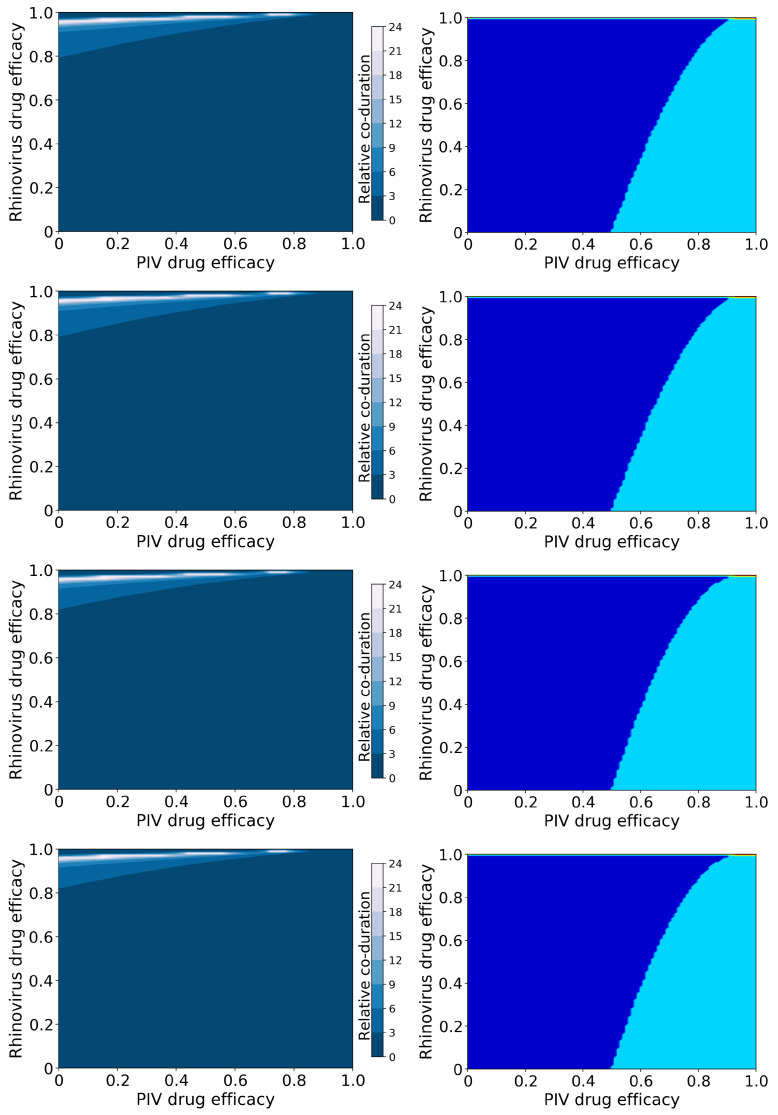
Coinfection durations and outcomes for treated rhinovirus and PIV coinfections. (**Left column**): Figures show the coinfection duration relative to an untreated coinfection where a value of 1 indicates that treatment has not changed the coinfection duration. (**Right column**): Figures show regions of different treatment outcomes: Dark blue indicates that both infections are detectable; light blue indicates that only hRV is detectable; yellow indicates that only PIV is detectable; and red indicates that both infections are suppressed. Figures show treatment with both antivirals reducing infection rate (**top row**); the rhinovirus antiviral reducing infection rate and the PIV antiviral reducing viral production (**second row**); the rhinovirus antiviral reducing viral production and the PIV antiviral reducing infection rate (**third row**); and both antivirals reducing viral production (**bottom row**).

As noted in the previous section, the broad treatment outcomes (Figure 6) here are independent of mechanism of action of the antivirals and are the same as for the acute coinfections simulated by the basic model, since the basic reproductive number is the same in all cases. Of interest here is how the coinfection duration changes with suboptimal treatment. Unlike IAV/RSV chronic coinfection, untreated hRV/PIV chronic coinfections have a finite coinfection duration because the chronic PIV load is below the level of detection. This means that it is possible to lengthen the coinfection duration in this case by treating the hRV infection with a high antiviral dose and treating PIV with a low antiviral dose. In this scenario, hRV is partially suppressed, giving PIV the opportunity to infect more cells and increase its chronic viral load, possibly above the threshold of infection. Thus, a “hidden” coinfection could be revealed by treatment of what appears to be a single viral infection.

## 4. Discussion

We used mathematical models to examine antiviral treatment of four different viral coinfection scenarios: Viruses with similar growth rates in acute or chronic coinfections, and viruses with very different growth rates in acute or chronic coinfections. We found that antiviral dose combinations that suppress one or both infections are independent of the mechanism of action of the antiviral and also do not depend on whether the coinfection is chronic or acute. Rather, suppression of one or both infections is determined by a reduction of the basic reproductive number below 1.

Antiviral mechanism of action and the type of coinfection do, however, affect how infection dynamics change with antiviral treatment that does not fully suppress infections. In particular, we found that it is possible to lengthen the coinfection duration with suboptimal treatment, or even to reveal hidden coinfections if antiviral treatment reduces the blocking effect of a dominant virus. Lengthening of infection durations with suboptimal treatment has been noted in other studies [42,55,68,73] and the dependence of infection duration on the antiviral mechanism of action has also been previously observed for single infections [42,73], therefore it is not unexpected to observe similar effects for viral coinfections. The implications of this for treatment of coinfections, however, can be more consequential than for a single infection, particularly when a previously suppressed infection is allowed to replicate. For example, it has been suggested that SARS-CoV-2 can be suppressed by other, typically more benign, respiratory viruses [74]. If we treat the other respiratory virus without simultaneously treating SARS-CoV-2 with a sufficiently high dose, the outcome for the patient might be worse than if the coinfection had been untreated.

While this study suggests that the antiviral mechanism of action and dose need to be carefully considered when treating coinfections, there are practical limitations that will play into clinical treatment decisions. There are not necessarily antivirals available for all respiratory infections, and if there are antivirals, they might not use one of the mechanisms of action examined here. For example, there are a number of antivirals for influenza [75], however none are approved yet for RSV. This study suggests that treating influenza without also treating the RSV could lead to longer RSV infections with a higher RSV viral load, therefore it might currently be better to not treat the influenza until a suitable RSV antiviral is available. Additionally, the effectiveness of currently available antivirals needs to be considered. We observed complete suppression of the coinfection only for high efficacies (high doses) of both antivirals. Depending on the toxicity or side effects of the antivirals, and any possible exacerbating effects of combining antivirals, it might not be possible to give patients the high doses needed to suppress both infections. In this case, studies such as these provide guidance on whether treatment with a suboptimal dose will make the coinfection worse.

It is important to remember that this study models an idealized scenario: Both infections are initiated at the same time with the same amount of virus and treatment is initiated at the time of infection. In reality, patients are likely infected with the two viruses sequentially, which changes the dynamics of the resulting coinfection [27,30,57,58]. Initial inoculum is also known to alter the dynamics of single infections [76,77,78] and can change the outcome of coinfections [57]. Similarly, treatment initiated after the onset of infection has a different effect on infection duration than prophylactic treatment [55,68,73], with effective antiviral treatment really only being possible if treatment is initiated before the time of viral titer peak [68]. Further modeling studies can help to provide guidance for these more realistic scenarios, but unfortunately, we often do not know when patients became infected with a virus, making treatment decisions very complicated.

Despite the limited scope of the respiratory virus coinfection scenarios examined in this manuscript, our results highlight some of the factors that need to be considered when making treatment decisions for viral coinfections. In particular, we have found that lengthening of coinfection duration is dependent on the antiviral mechanism of action and that possible treatment outcomes depend on the details of which viruses are involved in the coinfection.

## Figures and Tables

**Table 1:** Parameter values used for each virus. Taken from [57].

Virus	β	*k*	δ	*p*	*c*
	(PFU/mL)${}^{-1}$$\;\cdot \;d^{-1}$	$d^{-1}$	$d^{-1}$	(PFU/mL)$\;\cdot \;d^{-1}$	$d^{-1}$
IAV	8.27$\times 10^{-6}$	4.20	4.20	1.20$\times 10^{8}$	4.03
RSV	0.0308	1.27	1.27	7.65$\times 10^{3}$	1.27
hRV	2.06$\times 10^{-6}$	0.937	50.5	8.10$\times 10^{9}$	0.920
PIV	4.82$\times 10^{-7}$	13.2	13.2	2.12$\times 10^{8}$	0.567

## Data Availability

The data presented in this study are available in the manuscript and the Supplementary Material.

## Supplementary Materials

The following are available online at https://www.mdpi.com/article/10.3390/epidemiologia3010008/s1, File S1: Additional figures.

## Abbreviations

The following abbreviations are used in this manuscript:

hMPV	Human metapneumovirus
hRV	Human rhinovirus
IAV	Influenza A virus
PFU	Plaque forming unit
PIV	Parainfluenza virus
RSV	Respiratory syncytial virus
SARS-CoV-2	Severe acute respiratory syndrome coronavirus 2
