# Supplementary material for: Treatment of Respiratory Viral Coinfections
Source: Epidemiologia (Basel). 2022 Feb 23;3(1):81–96. doi: 10.3390/epidemiologia3010008 (PMC9620919; doi:10.3390/epidemiologia3010008)
Supplement: Supplementary file 1 [file epidemiologia-03-00008-s001.zip › epidemiologia-1509260-supplementary.pdf]

epidemiologia

MDPI

Supplementary Materials: Treatment of Respiratory Viral Coinfections

Paul Alexander and Hana Dobrovolny

1. Basic coinfection model

This section contains the individual infection durations and viral titer peak measurements predicted for treated coinfections by the basic coinfection model.

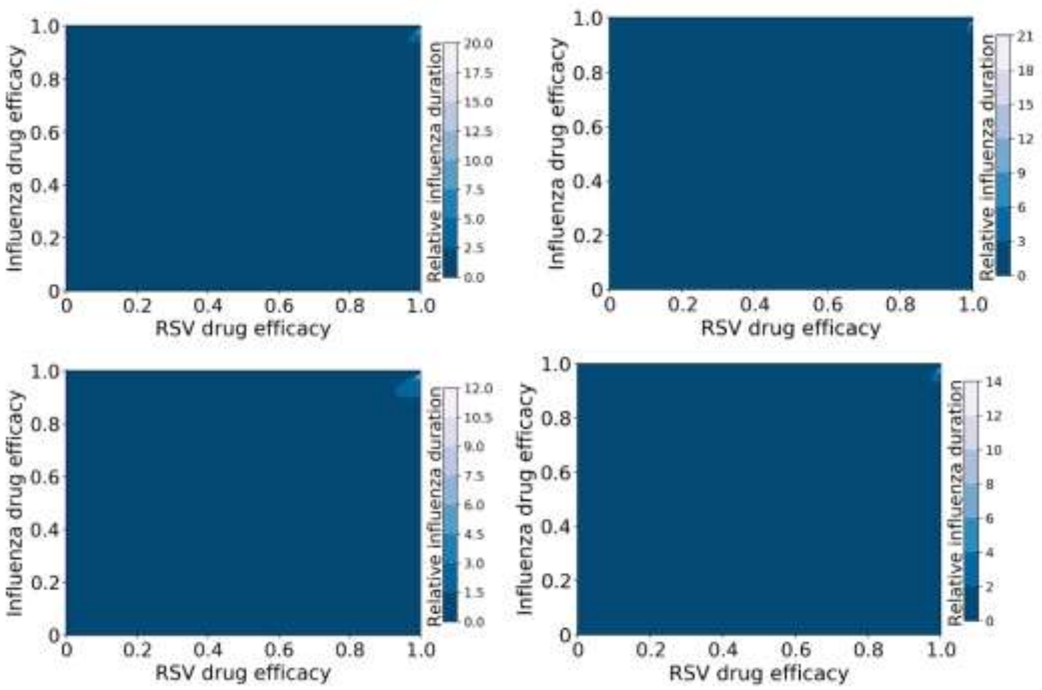

Figure S1. Duration of influenza infection for treated influenza and RSV coinfections. Figures show the duration of influenza infection relative to an untreated coinfection where a value of 1 indicates that treatment has not changed the influenza duration. Figures show treatment with both antivirals reducing infection rate (top left); the influenza antiviral reducing infection rate and the RSV antiviral reducing viral production (top right); the influenza antiviral reducing viral production and the RSV antiviral reducing infection rate (bottom left); and both antivirals reducing viral production (bottom right).

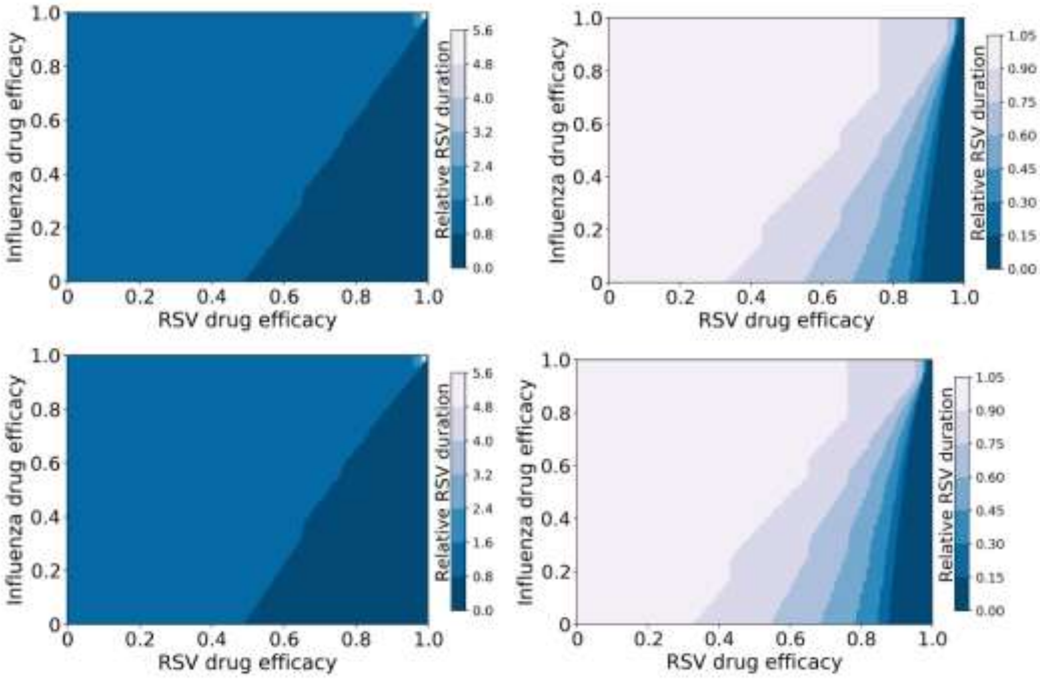

Figure S2. Duration of RSV infection for treated influenza and RSV coinfections. Figures show the duration of RSV infection relative to an untreated coinfection where a value of 1 indicates that treatment has not changed the RSV duration. Figures show treatment with both antivirals reducing infection rate (top left); the influenza antiviral reducing infection rate and the RSV antiviral reducing viral production (top right); the influenza antiviral reducing viral production and the RSV antiviral reducing infection rate (bottom left); and both antivirals reducing viral production (bottom right).

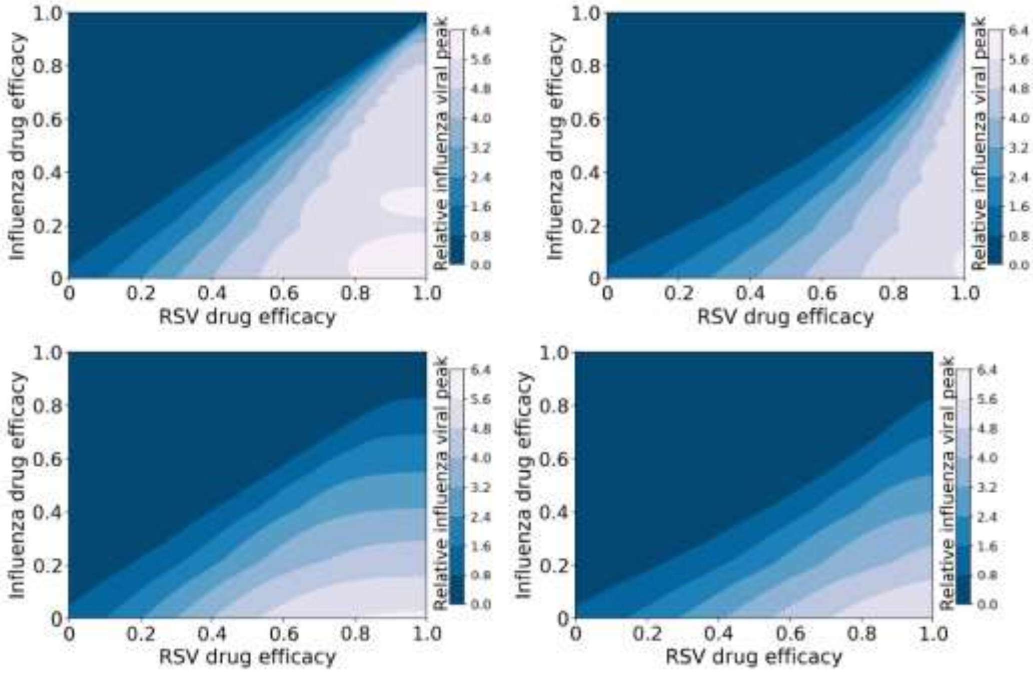

Figure S3. Influenza viral titer peak for treated influenza and RSV coinfections. Figures show the influenza viral titer peak relative to an untreated coinfection where a value of 1 indicates that treatment has not changed the peak viral load. Figures show treatment with both antivirals reducing infection rate (top left); the influenza antiviral reducing infection rate and the RSV antiviral reducing viral production (top right); the influenza antiviral reducing viral production and the RSV antiviral reducing infection rate (bottom left); and both antivirals reducing viral production (bottom right).

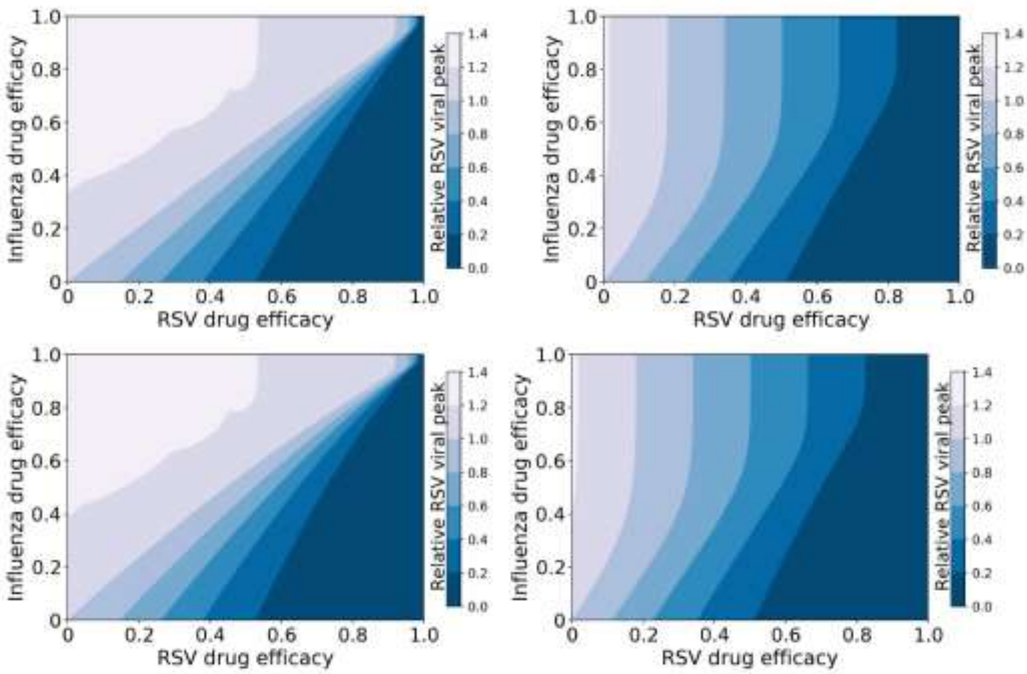

Figure S4. RSV viral titer peak for treated influenza and RSV coinfections. Figures show the RSV viral titer peak relative to an untreated coinfection where a value of 1 indicates that treatment has not changed the peak viral load. Figures show treatment with both antivirals reducing infection rate (top left); the influenza antiviral reducing infection rate and the RSV antiviral reducing viral production (top right); the influenza antiviral reducing viral production and the RSV antiviral reducing infection rate (bottom left); and both antivirals reducing viral production (bottom right).

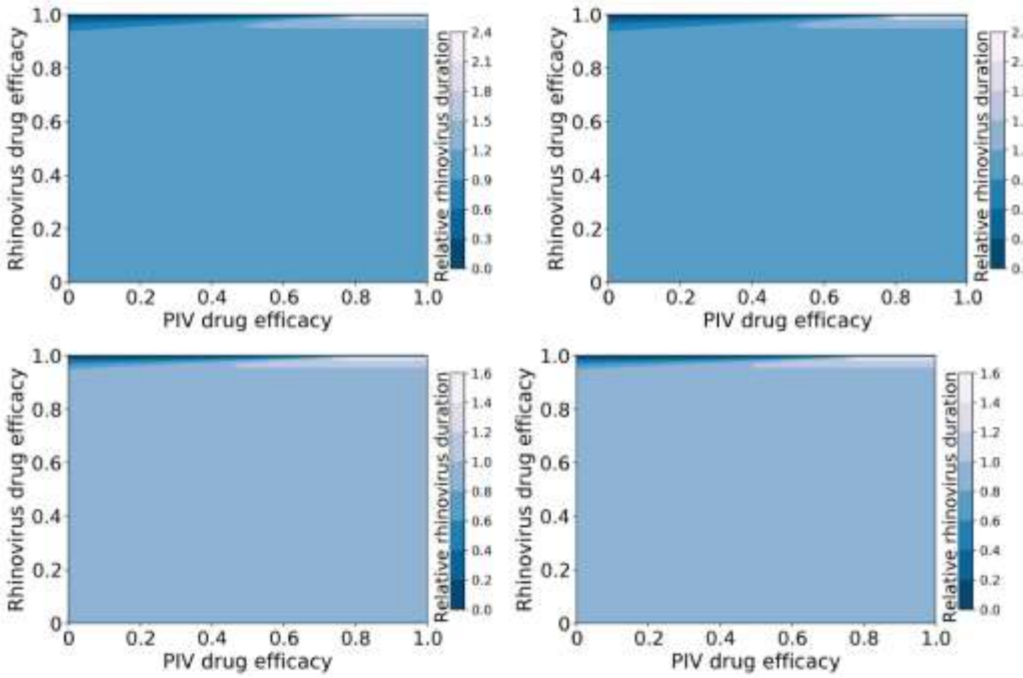

Figure S5. Duration of rhinovirus infection for treated rhinovirus and PIV coinfections. Figures show the duration of rhinovirus infection relative to an untreated coinfection where a value of 1 indicates that treatment has not changed the rhinovirus duration. Figures show treatment with both antivirals reducing infection rate (top left); the rhinovirus antiviral reducing infection rate and the PIV antiviral reducing viral production (top right); the rhinovirus antiviral reducing viral production and the PIV antiviral reducing infection rate (bottom left); and both antivirals reducing viral production (bottom right).

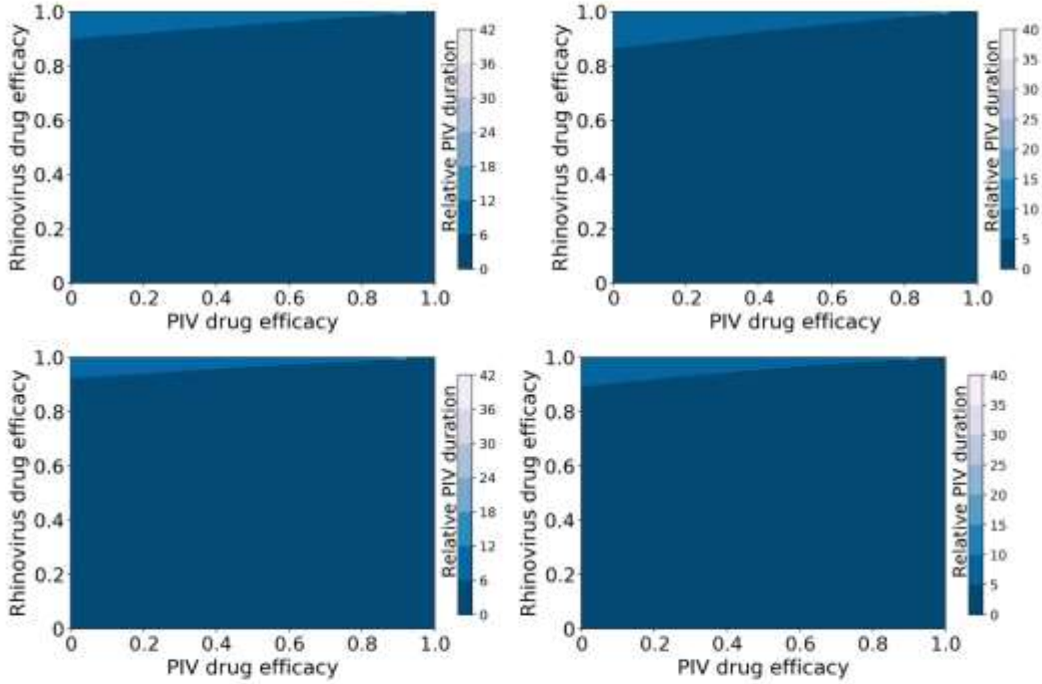

Figure S6. Duration of PIV infection for treated rhinovirus and PIV coinfections. Figures show the duration of PIV infection relative to an untreated coinfection where a value of 1 indicates that treatment has not changed the PIV duration. Figures show treatment with both antivirals reducing infection rate (top left); the rhinovirus antiviral reducing infection rate and the PIV antiviral reducing viral production (top right); the rhinovirus antiviral reducing viral production and the PIV antiviral reducing infection rate (bottom left); and both antivirals reducing viral production (bottom right).

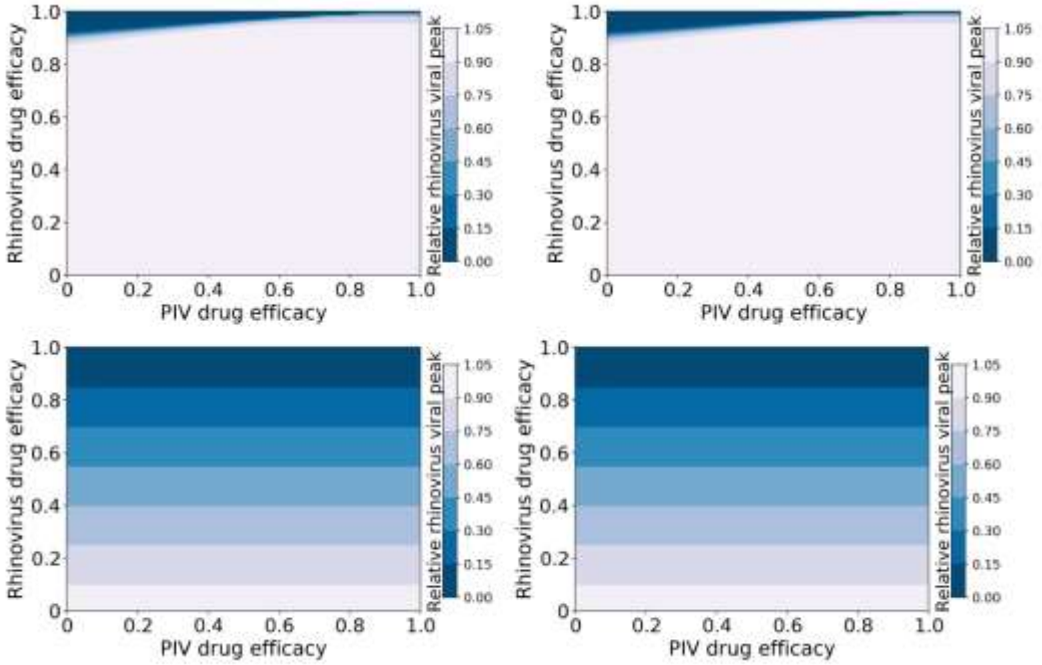

Figure S7. Rhinovirus viral titer peak for treated rhinovirus and PIV coinfections. Figures show the rhinovirus viral titer peak relative to an untreated coinfection where a value of 1 indicates that treatment has not changed the peak viral load. Figures show treatment with both antivirals reducing infection rate (top left); the rhinovirus antiviral reducing infection rate and the PIV antiviral reducing viral production (top right); the rhinovirus antiviral reducing viral production and the PIV antiviral reducing infection rate (bottom left); and both antivirals reducing viral production (bottom right).

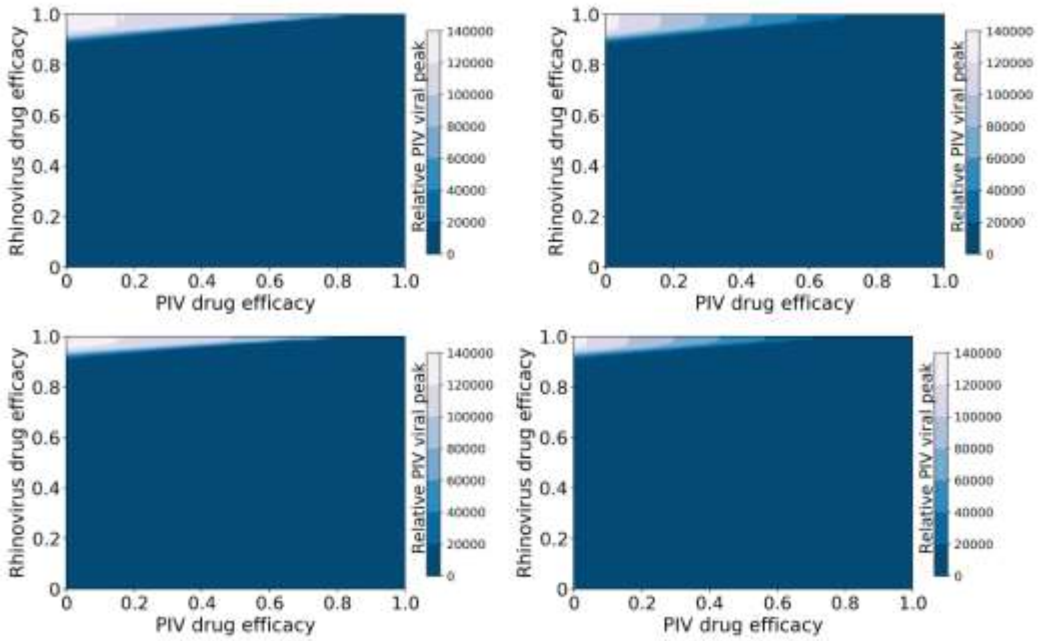

Figure S8. PIV viral titer peak for treated rhinovirus and PIV coinfections. Figures show the PIV viral titer peak relative to an untreated coinfection where a value of 1 indicates that treatment has not changed the peak viral load. Figures show treatment with both antivirals reducing infection rate (top left); the rhinovirus antiviral reducing infection rate and the PIV antiviral reducing viral production (top right); the rhinovirus antiviral reducing viral production and the PIV antiviral reducing infection rate (bottom left); and both antivirals reducing viral production (bottom right).

2. Superinfection model

This section contains the individual infection durations and viral titer peak measurements predicted for treated coinfections by the superinfection model.

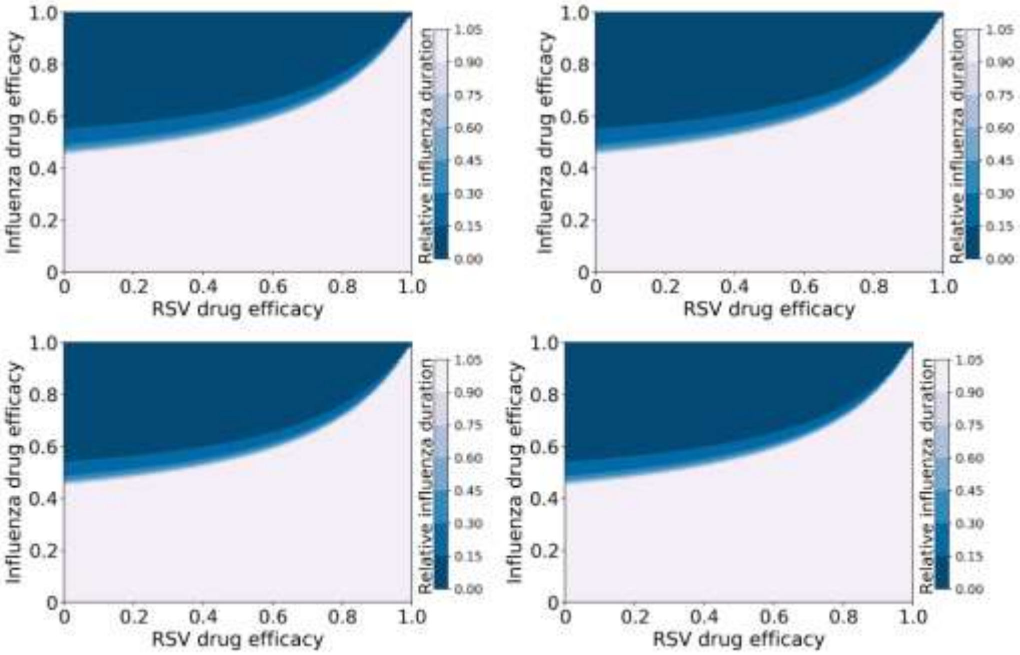

Figure S9. Duration of influenza infection for treated influenza and RSV coinfections. Figures show the duration of influenza infection relative to an untreated coinfection where a value of 1 indicates that treatment has not changed the influenza duration. Figures show treatment with both antivirals reducing infection rate (top left); the influenza antiviral reducing infection rate and the RSV antiviral reducing viral production (top right); the influenza antiviral reducing viral production and the RSV antiviral reducing infection rate (bottom left); and both antivirals reducing viral production (bottom right).

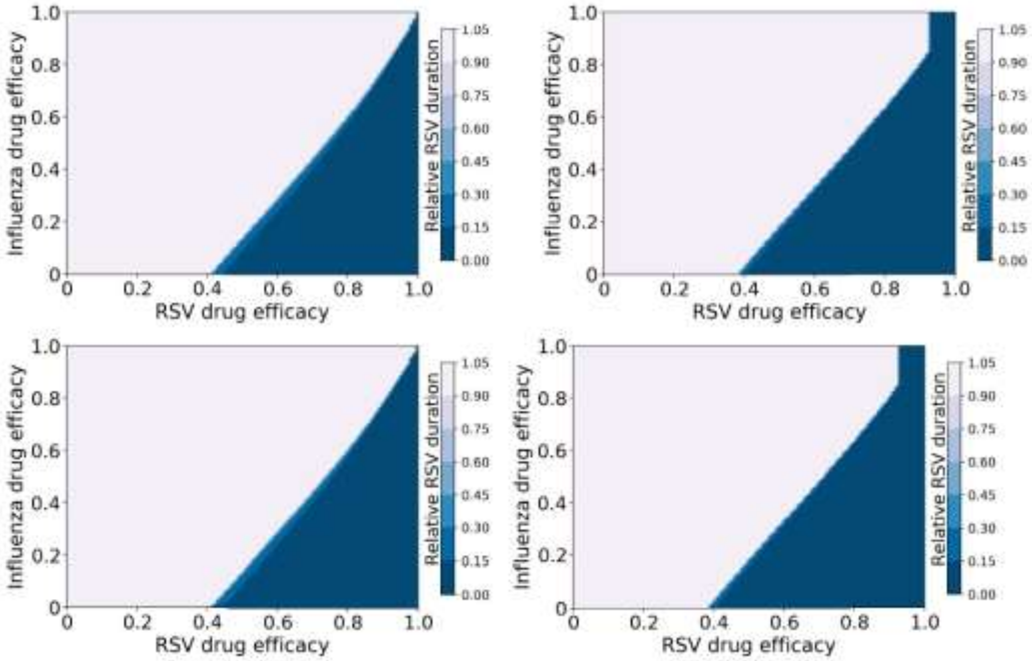

Figure S10. Duration of RSV infection for treated influenza and RSV coinfections. Figures show the duration of RSV infection relative to an untreated coinfection where a value of 1 indicates that treatment has not changed the RSV duration. Figures show treatment with both antivirals reducing infection rate (top left); the influenza antiviral reducing infection rate and the RSV antiviral reducing viral production (top right); the influenza antiviral reducing viral production and the RSV antiviral reducing infection rate (bottom left); and both antivirals reducing viral production (bottom right).

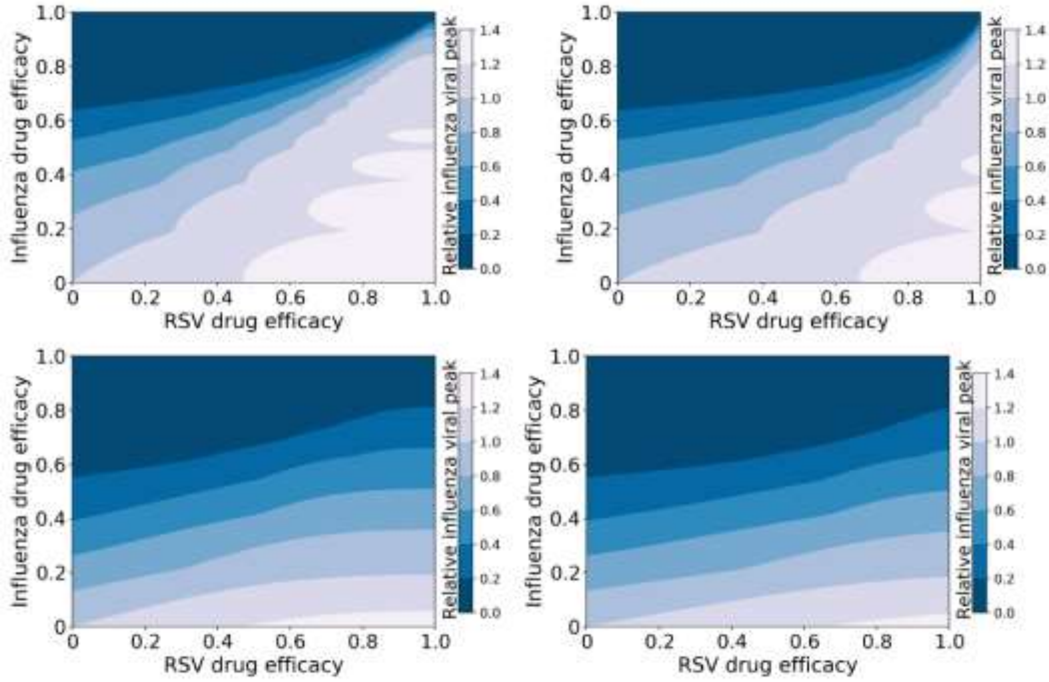

Figure S11. Influenza viral titer peak for treated influenza and RSV coinfections. Figures show the influenza viral titer peak relative to an untreated coinfection where a value of 1 indicates that treatment has not changed the peak viral load. Figures show treatment with both antivirals reducing infection rate (top left); the influenza antiviral reducing infection rate and the RSV antiviral reducing viral production (top right); the influenza antiviral reducing viral production and the RSV antiviral reducing infection rate (bottom left); and both antivirals reducing viral production (bottom right).

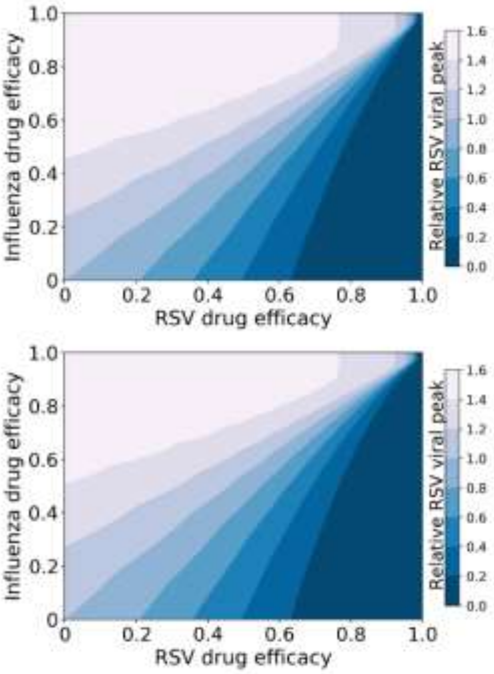

Figure S12. RSV viral titer peak for treated influenza and RSV coinfections. Figures show the RSV viral titer peak relative to an untreated coinfection where a value of 1 indicates that treatment has not changed the peak viral load. Figures show treatment with both antivirals reducing infection rate (top left); the influenza antiviral reducing infection rate and the RSV antiviral reducing viral production (top right); the influenza antiviral reducing viral production and the RSV antiviral reducing infection rate (bottom left); and both antivirals reducing viral production (bottom right).

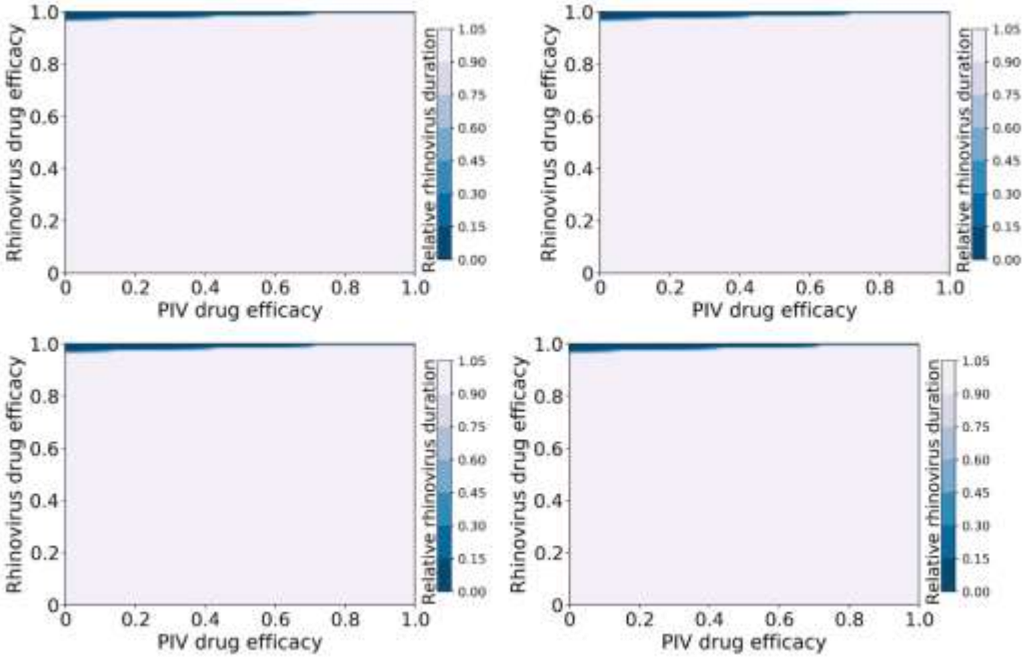

Figure S13. Duration of rhinovirus infection for treated rhinovirus and PIV coinfections. Figures show the duration of rhinovirus infection relative to an untreated coinfection where a value of 1 indicates that treatment has not changed the rhinovirus duration. Figures show treatment with both antivirals reducing infection rate (top left); the rhinovirus antiviral reducing infection rate and the PIV antiviral reducing viral production (top right); the rhinovirus antiviral reducing viral production and the PIV antiviral reducing infection rate (bottom left); and both antivirals reducing viral production (bottom right).

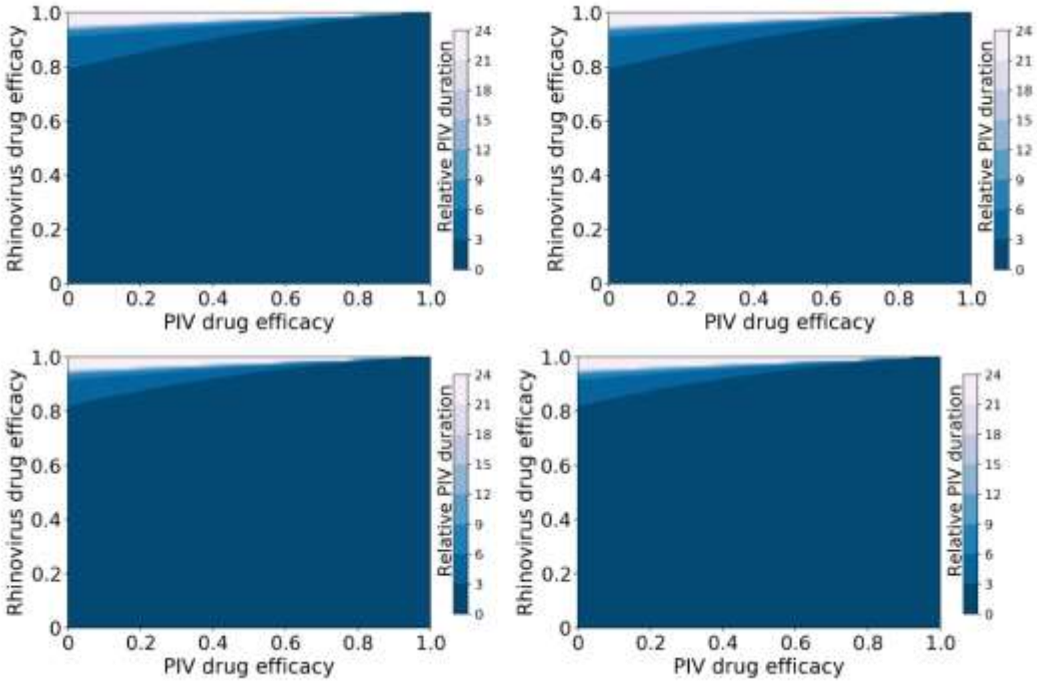

Figure S14. Duration of PIV infection for treated rhinovirus and PIV coinfections. Figures show the duration of PIV infection relative to an untreated coinfection where a value of 1 indicates that treatment has not changed the PIV duration. Figures show treatment with both antivirals reducing infection rate (top left); the rhinovirus antiviral reducing infection rate and the PIV antiviral reducing viral production (top right); the rhinovirus antiviral reducing viral production and the PIV antiviral reducing infection rate (bottom left); and both antivirals reducing viral production (bottom right).

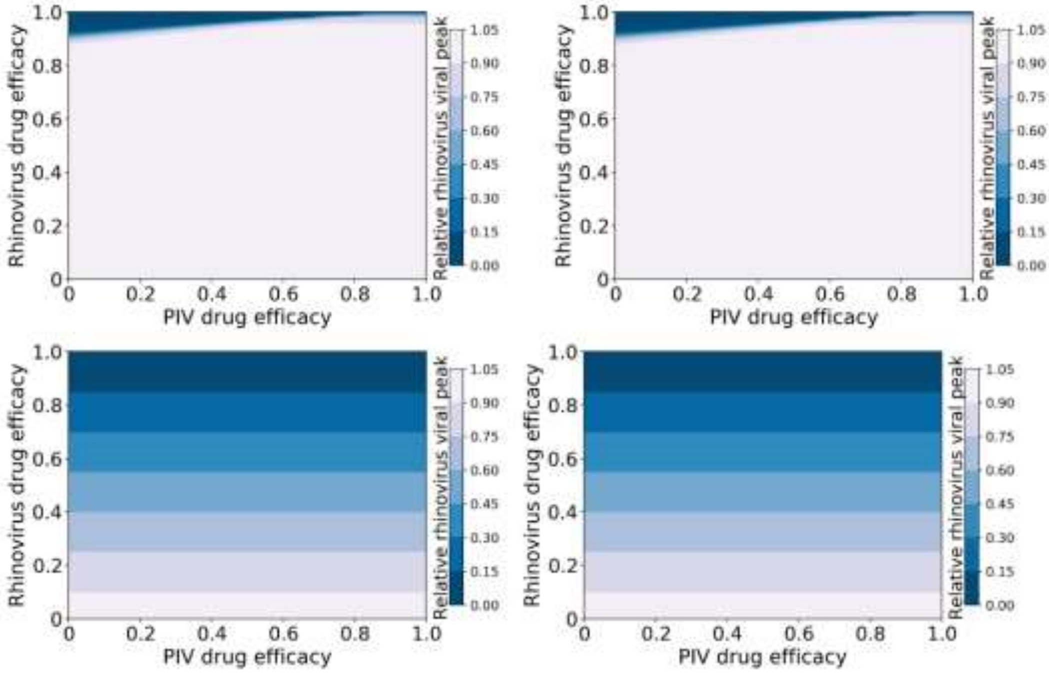

Figure S15. Rhinovirus viral titer peak for treated rhinovirus and PIV coinfections. Figures show the rhinovirus viral titer peak relative to an untreated coinfection where a value of 1 indicates that treatment has not changed the peak viral load. Figures show treatment with both antivirals reducing infection rate (top left); the rhinovirus antiviral reducing infection rate and the PIV antiviral reducing viral production (top right); the rhinovirus antiviral reducing viral production and the PIV antiviral reducing infection rate (bottom left); and both antivirals reducing viral production (bottom right).

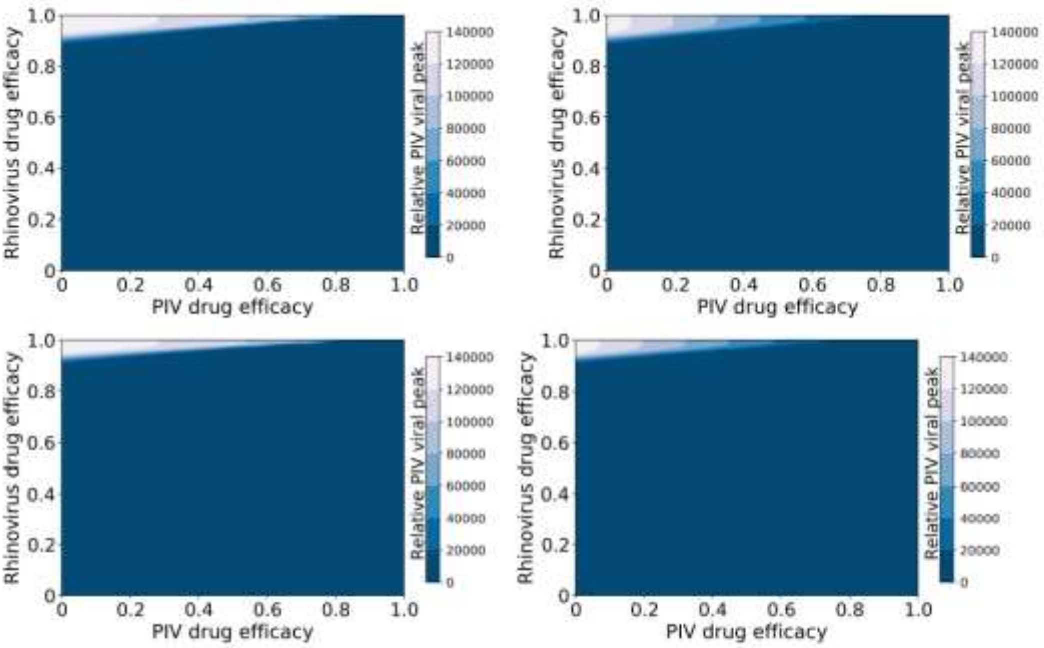

Figure S16. PIV viral titer peak for treated rhinovirus and PIV coinfections. Figures show the PIV viral titer peak relative to an untreated coinfection where a value of 1 indicates that treatment has not changed the peak viral load. Figures show treatment with both antivirals reducing infection rate (top left); the rhinovirus antiviral reducing infection rate and the PIV antiviral reducing viral production (top right); the rhinovirus antiviral reducing viral production and the PIV antiviral reducing infection rate (bottom left); and both antivirals reducing viral production (bottom right).
